# Targeted neuroplasticity in spatiotemporally patterned invasive neuromodulation therapies for improving clinical outcomes

**DOI:** 10.3389/fninf.2023.1150157

**Published:** 2023-03-24

**Authors:** Anders J. Asp, Yaswanth Chintaluru, Sydney Hillan, J. Luis Lujan

**Affiliations:** ^1^Mayo Clinic Graduate School of Biomedical Sciences, Mayo Clinic, Rochester, MN, United States; ^2^Department of Neurologic Surgery, Mayo Clinic, Rochester, MN, United States; ^3^Department of Neurology and Neurosurgery, University of Colorado Anschutz School of Medicine, Aurora, CO, United States; ^4^Department of Physiology and Biomedical Engineering, Mayo Clinic, Rochester, MN, United States

**Keywords:** neuroplasticity, spike timing dependant plasticity, deep brain stimulation (DBS), biofeedback, network control, transcranial magnetic stimulation (TMS), neuromodulation, neurosurgery

## Introduction

Invasive neuromodulation is routinely used to effectively treat the symptoms of movement (Dallapiazza et al., 2019; Limousin and Foltynie, 2019) and psychiatric (Visser-Vandewalle et al., 2022) disorders with high success despite a limited understanding of their mechanisms of action. While the distinct neuroanatomical targets that are stimulated vary depending on the condition being treated and any existing comorbidities, the predominant neuromodulation strategy is to apply a fixed-frequency electrical current to the corresponding neural targets for symptom relief. In the case of movement disorders such as Parkinson's disease (PD), symptom reduction manifests within seconds or minutes following stimulation onset and disappears within a similar time course following the cessation of stimulation (Hristova et al., 2000; Temperli et al., 2003; Ducharme et al., 2011; Pugh, 2019). Maladaptive neuroplasticity, defined as plasticity underlying a disruption in normal neural network function, contributes to numerous neurologic and psychiatric conditions such as chronic pain (Kuner and Flor, 2017), mood disorders (Duman, 2002), movement disorders (McPherson et al., 2015; Li, 2017; Seeman et al., 2017; Peng et al., 2018; Versace et al., 2018; Madadi Asl et al., 2022), tinnitus (Engineer et al., 2011), addiction (Kauer and Malenka, 2007; Kalivas and O'Brien, 2008; Famitafreshi and Karimian, 2019), and depression (Duman et al., 2016). While some invasive neuromodulation approaches treat this underlying neuroplasticity (Creed et al., 2015; McPherson et al., 2015; Seeman et al., 2017; Peng et al., 2018; Versace et al., 2018; Asl et al., 2023), most do not. Thus, the neuromodulation community must consider well-characterized biophysical phenomena such as synaptic plasticity as inspiration when developing next-generation neuromodulation therapies rather than re-applying stimulation paradigms designed for movement disorders to improve treatment outcomes in all conditions, such as psychiatric disorders.

### Targeted neuroplasticity as a tool to treat neurologic and psychiatric indications

Targeted neuroplasticity encompasses neuromodulation approaches designed to induce and maintain a long-term influence over nervous system function through long-term potentiation (LTP) or long-term depression (LTD) such that symptom improvement persists after stimulation cessation. Examples of non-invasive neuromodulation approaches that maintain targeted neuroplasticity include transcranial magnetic stimulation (TMS) (Horvath et al., 2010; Valero-Cabré et al., 2017) and vibrotactile coordinated reset (CR) (Syrkin-Nikolau et al., 2018; Pfeifer et al., 2021). These approaches contrast with some conventional invasive neuromodulation approaches such as fixed-frequency deep brain stimulation (DBS), in which acute symptoms are managed only during stimulation (Herrington et al., 2015; Ashkan et al., 2017; Pugh, 2019).

**Figure 1:**
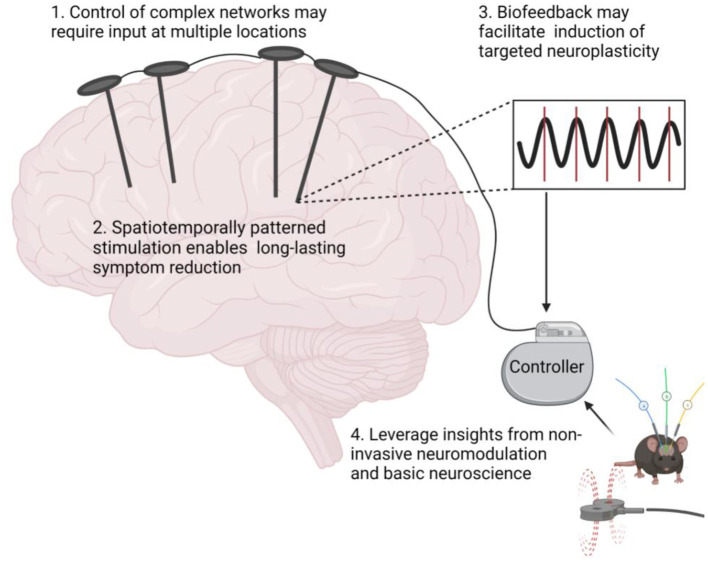
Targeted neuroplasticity approaches for invasive neuromodulation therapies. Figure created with Biorender.com.

Here, we postulate that targeted neuroplasticity through spatiotemporally patterned stimulation may improve clinical outcomes and enhance invasive therapies such as DBS by reversing maladaptive plasticity rather than treating symptoms. To this end, we propose four considerations for incorporating targeted neuroplasticity into invasive neuromodulation therapies (Figure 1).

### Control of complex networks requires spatiotemporally precise stimulation at multiple network locations to improve clinically significant long-term symptom reduction

Neurologic conditions are often associated with neural network dysfunction (Spencer, 2002; Palop et al., 2006; Rosin et al., 2007), and as such, clinically-effective outcomes require timely interventions at multiple network locations (Tu et al., 2018). While initial studies suggested neural activity could be altered from a single node (Gu et al., 2015), the interconnected topology of neural networks complicates selection of a single control node from which to apply stimulation. Furthermore, multiple studies using functional magnetic resonance imaging (fMRI) and other techniques have demonstrated that stimulation at multiple nodes enhances network control (Capotosto et al., 2014; Fox et al., 2014; Pasqualetti et al., 2014; Tu et al., 2018). More importantly, studies have shown that enhanced multi-node network controllability can be achieved *via* paired stimulation of multiple connected brain regions such as inter-hemispheric dPM-M1 cortex (Lafleur et al., 2016). A clear example of this concept is the use of dual-site DBS placed in the centromedian-parafascicular complex and ventral capsule/ventral striatum to effectively treat motor and non-motor symptoms of severe, medication-resistant Tourette syndrome (Kakusa et al., 2019). Studies thus suggest that multi-location stimulation may improve control of pathological network function underlying symptoms.

#### Spatiotemporally patterned stimulation enables long-lasting desynchronization of pathological network activity and sustained symptom reduction

Spatiotemporally patterned stimulation has distinct advantages over traditional high-frequency (>100 Hz) stimulation such as facilitation of long-lasting targeted neuroplasticity and desynchronization of pathological network activity leading to symptom reduction. The relative timing between presynaptic and postsynaptic activation influences synaptic strength through a mechanism known as spike-timing dependent plasticity (STDP), is known to profoundly influence brain network function through changes in the direction and magnitude of synaptic strength (Markram et al., 1997; Bi and Poo, 1998; Dan and Poo, 2004; Caporale and Dan, 2008; Brzosko et al., 2019). STDP mechanisms are leveraged by emerging spatiotemporally patterned neuromodulation approaches such as decoupling time-shifted stimulation (Kromer and Tass, 2020; Asl et al., 2023) periodic multichannel stimulation (Kromer and Tass, 2022), and CR (Pfister et al., 2010). These therapies facilitate long-lasting desynchronization of pathologically coherent network activity underlying conditions like Parkinson's Disease (PD) by applying spatiotemporally patterned electric stimulation across subcortical targets such as the STN (Tass, 2003; Tass and Majtanik, 2006; Pfister et al., 2010; Adamchic et al., 2014; Ebert et al., 2014; Wang et al., 2016; Madadi Asl et al., 2018). From a therapeutic standpoint, a major benefit of spatiotemporally patterned therapies is that discontinuous and lower frequency stimulation may reduce the risk of side effects attributable to chronic continuous stimulation (Ferraye et al., 2008; Xie et al., 2012). Furthermore, therapies such as CR demonstrate sustained symptom reduction after stimulation cessation (Tass et al., 2012; Adamchic et al., 2017; Syrkin-Nikolau et al., 2018; Ho et al., 2021; Pfeifer et al., 2021; Wang et al., 2022). Similarly, paired phase-locked stimulation of the infralimbic cortex and basolateral amygdala alters synaptic strength and theta band coherence in a manner that that persists after stimulation cessation (Lo et al., 2020).

Numerous studies achieve targeted neuroplasticity with spatiotemporally patterned stimulation delivered across multiple stimulation modalities. For example, repeated pairing of low frequency (0.1 Hz) DBS with TMS of M1-cortex alters corticostriatal plasticity in humans (Udupa et al., 2016). Similarly, the application of transcranial direct or alternating current stimulation prior to TMS has been shown to alter the effectiveness of the TMS-based plasticity induction protocol (Cosentino et al., 2012; Guerra et al., 2018; Nakazono et al., 2021). Additionally, pairing DBS of midbrain locomotor regions with epidural stimulation of the lumbar spinal cord improves motor function in a rat model of spinal cord injury (Bonizzato et al., 2021). One clinical case report found improved motor function in a patient with multiple system atrophy and predominant parkinsonism when bilateral subthalamic nucleus (STN) DBS and spinal cord stimulation were combined (Li et al., 2022). Taken together, these examples demonstrate that spatiotemporally patterned simulation may enable long-lasting reductions in symptoms and side effects and expand invasive neuromodulation indications while improving power consumption efficiency.

#### Biofeedback may facilitate induction of targeted neuroplasticity

Closed-loop neuromodulation approaches leverage biofeedback to guide stimulation parameter selection in a wide range of circuitopathies underlying conditions such as epilepsy (Seitz, 2013), PD (Kühn et al., 2009; Weinberger et al., 2012), essential tremor (Thompson et al., 2014), and dystonia (Barow et al., 2014), in which oscillation frequency abnormalities serve as biomarkers that can inform stimulation parameter selection to improve symptom reduction (Thompson et al., 2014). For example, electrophysiological activity recorded during electrographic seizures can trigger DBS to interrupt seizure progression (Thomas and Jobst, 2015; Razavi et al., 2020). Furthermore, studies indicate that phase-aligned stimulation triggered by local field potentials can alter pathological cortical-striatal-pallidal activity and cortico-amygdalar coherence, reducing symptoms of obsessive-compulsive disorder (OCD) (Olsen et al., 2020) and anxiety (Lo et al., 2020), respectively. Stimulation of the ventrolateral (VL) thalamus aligned to patients' limb tremor reduces tremor severity in essential tremor patients through a mechanism involving STDP (Cagnan et al., 2017). Thus, initial exploration of closed-loop stimulation as a mechanism to achieve targeted neuroplasticity promises to be a versatile tool in the treatment of neurologic disease and injury. As such, an expanded investigation of targeted neuroplasticity that incorporates biofeedback measurements may expand this powerful technique into a readily translatable clinical treatment.

#### Insights from non-invasive neuromodulation and basic neuroscience may inform novel invasive targeted neuroplasticity approaches

Non-invasive neuromodulation therapies such as TMS or focused ultrasound have embraced the targeted neuroplasticity philosophy out of necessity. The immobile nature of non-invasive systems, frequently due to large size and cost of the necessary hardware (Horvath et al., 2010; Anderson et al., 2012; Santarnecchi et al., 2018; Carmi et al., 2019; Mehta et al., 2019; Sabbagh et al., 2020), has necessitated the development of stimulation protocols designed to induce long-term plastic changes in brain function. Consequently, numerous non-invasive stimulation protocols have been designed to facilitate long-term changes in neuroplasticity (Todd et al., 2010; Bunday and Perez, 2012; Jacobs et al., 2012; Urbin et al., 2017; Aftanas et al., 2018; Kozyrev et al., 2018). Despite being limited to engaging cortical targets at a poor spatial specificity on the order of 1,000 mm^2^ (van de Ruit and Grey, 2016), TMS has succeeded where more precise invasive approaches such as DBS have failed (e.g., treatment-resistant major depressive disorder). It is thus surprising that few studies are seeking to translate FDA-approved non-invasive plasticity-inducing stimulation protocols to invasive techniques such as DBS, which offer a more selective target engagement and, therefore, fewer side effects (Ni et al., 2019).

Adapting classical neuroplasticity induction protocols rooted in basic neuroscience may form the foundation for novel therapies for treatment-resistant clinical indications. An example where DBS has produced less-than-satisfactory results is in the treatment of Alzheimer's disease. A randomized, sham-controlled, double-blinded clinical trial of patients with Alzheimer's disease found continuous high frequency (130 Hz) DBS of the fornix, a brain region implicated in learning and memory (Douet and Chang, 2015), does not improve cognitive function (Lozano et al., 2016). Theta burst microstimulation (5 pulses separated by 200 ms, 100 Hz) is a well-described plasticity induction protocol established *ex vivo* to cause LTP in neural circuits (Abrahamsson et al., 2016). Theta burst stimulation of the right entorhinal cortex significantly increased performance on pattern separation and memory recall, suggesting utility for the treatment of Alzheimer's disease (Titiz et al., 2017). Moreover, intermittent theta-burst stimulation results in safe and reliable changes in dorsolateral prefrontal cortex electrophysiology (Bentley et al., 2020) and may improve treatment of neurological conditions with historically poor success rates. Emerging optogenetics-inspired DBS protocols consisting of 1 Hz electrical stimulation of the Nucleus Accumbens paired with a D1-Dopamine receptor antagonist reverse behavioral adaptations in a rodent model of addiction (Creed et al., 2015). Similarly, brief bursts of electrical stimulation in the external Globus Pallidus enables control of distinct neuronal subpopulations and produces long-lasting therapeutic benefits in dopamine depleted mice (Spix et al., 2021). Taken together, targeted neuroplasticity induction protocols should be considered as an alternative to high-frequency stimulation to treat neurological conditions in which disease symptomology is predicated on maladaptive neuroplasticity.

## Discussion

A strong feature of traditional DBS is its reversibility, which led it to become a favorable alternative to lesioning procedures for treatment of neurologic and psychiatric disorders (Pugh, 2019). While targeted plasticity can be viewed as a shift away from a reversible surgical procedure, it must be noted that traditional DBS, such as STN DBS also causes changes in plasticity (Herrington et al., 2015; Melon et al., 2015; Chassain et al., 2016). However, high-frequency STN DBS does not create long-lasting neuroplastic changes that may support symptom reduction after cessation of stimulation, supporting the reversibility of DBS therapies (Pugh, 2019).

Interventions that provide long-term changes in targeted neuroplasticity through spatiotemporally patterned stimulation offer distinct advantages over traditional high-frequency invasive neuromodulation, chiefly the ability to manipulate underlying disease pathophysiology, persistent symptom improvement after stimulation cessation, reduced power consumption from lower stimulation frequencies, amplitudes, and duty cycles, and improved circuit specificity that minimizes off-target effects. Thus, targeted neuroplasticity approaches may enable expanded avenues for treatment of disorders associated with maladaptive plasticity, such as Tourette's syndrome (Nespoli et al., 2018), OCD (Kreitzer and Malenka, 2008; Maia et al., 2008), Schizophrenia (McCutcheon et al., 2019), PD (Shen et al., 2008; Kravitz et al., 2010; Parker et al., 2018), and Manic Depression (Lee et al., 2018).

Despite the advantages of leveraging targeted neuroplasticity in spatiotemporally patterned invasive neuromodulation therapies, there remain numerous barriers to clinical implementation. When considering the need for multi-nodal circuit control, it is paramount to consider that additional hardware may incur additional surgical risks (Chiong et al., 2018). However, multi-lead DBS procedures are safe and routinely performed (Dallapiazza et al., 2019). Non-invasive options such as TMS can be paired with invasive stimulation to decrease surgical risk of additional implants while enabling additional therapeutic approaches.

There remains a real risk that preclinical findings do not translate between species, particularly to humans (de Oliveira et al., 2021). Consequently, caution must be taken when applying plasticity induction protocols clinically. While application of any novel stimulation paradigm comes with risk, a reasonable starting point for translating a novel neuroplasticity induction protocol to humans is to test plasticity induction protocols in individuals with existing implanted pulse generators, particularly if the system is capable of electrophysiological monitoring. An example of this strategy is evident in the previously mentioned multi-modal approach, where TMS pulses were paired with electrical stimulation of previously indwelling STN DBS electrodes (Udupa et al., 2016). Testing plasticity protocols in such a manner enables feasibility testing in humans without risks inherent in *de novo* surgical procedures.

Considering the advantages of invasive over non-invasive neuromodulation approaches, we must ask the question, “Why is it that targeted neuroplasticity-inducing protocols such as those used by non-invasive therapies are not widely used invasive neuromodulation therapies?” Perhaps the immediately effective therapeutic benefits of invasive neuromodulation approaches unnecessarily constrain parameter selection. Rather than treat stimulation-induced synaptic plasticity as an obstacle that interferes with long-term efficacy of traditional high-frequency stimulation, stimulation-induced neuroplasticity should be considered as a therapeutic mechanism. This mechanism may be sensitive to numerous parameters, including the type of underlying synaptic plasticity, synaptic transmission delays, the spatiotemporal stimulation pattern, the stimuli shape, and stimulation context. Borrowing inspiration from the protocols of non-invasive neuromodulation like TMS, vibrotactile CR, and basic neuroscience may help improve the clinical outcomes of DBS by creating lasting symptom benefit while broadening the clinical indications that can be treated with invasive therapies.

Clinical invasive neuromodulation approaches have remained largely unchanged since their inception. For example, high-frequency DBS is still the gold standard for treating medically refractory movement disorders. However, neuromodulation is limited in its ability to relieve disease symptoms after stimulation cessation. Re-designing stimulation protocols to address the underlying pathophysiology of disease circuitopathies may improve the current treatment of disorders and expand clinical applications. Integrating this approach into stimulation protocols may require control of complex networks through input at multiple nodes, long-lasting desynchronization of pathologically coherent network activity for long-lasting symptom reduction, and insight from non-invasive neuromodulation and basic neuroscience. Thus, targeted neuroplasticity may pave new paths in neuromodulation, expanding indications and improving disease pathophysiology.

## Author contributions

AA and JL conceived of the idea. YC and AA wrote the manuscript. AA, YC, and SH prepared figures. All authors discussed the results and contributed to the final manuscript.
